# Pseudolycorine chloride ameliorates Th17 cell-mediated central nervous system autoimmunity by restraining myeloid-derived suppressor cell expansion

**DOI:** 10.1080/13880209.2022.2063344

**Published:** 2022-09-09

**Authors:** Gan Zhang, Xinying Zhu, Fan Yang, Juan Li, Xiao Leng, Chunfen Mo, Limei Li, Yantang Wang

**Affiliations:** a Clinical Laboratory, Clinical Medical College and The First Affiliated Hospital of Chengdu Medical College, Chengdu Medical College, Chengdu, China; b Department of Pharmacology, School of Pharmacy, Chengdu Medical College, Chengdu, China; c College of Pharmacy, Southwest Minzu University, Chengdu, China

**Keywords:** MDSCs, M-MDSCs, experimental autoimmune encephalomyelitis/multiple sclerosis, immunosuppression, IL-17A

## Abstract

**Context:**

The alkaloids of *Narcissus tazetta* L. var. *Chinensis* Roem (Amaryllidaceae) have antitumor and antiviral activities. However, the immunopharmacological effects of one of its constituents, pseudolycorine chloride (PLY), have not been reported yet.

**Objective:**

We evaluated the effect of PLY on myeloid-derived suppressor cells (MDSCs) expansion and differentiation into monocyte-like MDSCs (M-MDSCs) and examined whether PLY alleviates Th17 cell-mediated experimental autoimmune encephalomyelitis (EAE), a murine model of multiple sclerosis (MS).

**Materials and methods:**

*In vitro*, MDSCs were treated with PLY (0.67, 2 and 6 μM) or solcitinib (10 μM, positive control) for 48 or 96 h, and their proliferation, expansion, and differentiation into M-MDSCs were examined by flow cytometry. Myelin oligodendrocyte glycoprotein (MOG_35–55_) was used to induce EAE in female C57BL/6 mice, and the mice were treated with 40 mg/kg/d PLY or 1 mg/kg/d FK-506 (tacrolimus, positive control) for 21 days. Inflammatory infiltration, spinal cord demyelination, and MDSCs and Th17 cells infiltration into the spinal cord were examined using haematoxylin and eosin staining, Luxol fast blue staining, and immunofluorescence, respectively.

**Results:**

*In vitro*, PLY (IC50/24 h = 6.18 μM) significantly inhibited IL-6 and GM-CSF-induced MDSCs proliferation, expansion and differentiation into M-MDSCs at all concentrations used. However, these concentrations did not show cytotoxicity. In mice, PLY (40 mg/kg) treatment alleviated EAE and inhibited inflammatory infiltration, demyelination, and MDSCs and Th17 cells infiltration into the spinal cord.

**Discussion and conclusions:**

PLY may be an excellent candidate for the treatment of MS and other autoimmune diseases.

## Introduction

Myeloid-derived suppressor cells (MDSCs) are derived from common myeloid progenitors, and their expansion and differentiation are supported by the myeloid-specific growth factors GM-CSF, G-CSF, M-CSF and the pro-inflammatory cytokines IL-6 and IL-11. MDSCs were originally identified as CD11b^+^Gr-1^hi^ cells in tumour-bearing mice. This classical cell population can be further subdivided into granulocyte-like MDSCs (Ly6C^med^Ly6G^+^, G-MDSCs) and monocyte-like MDSCs (Ly6C^+^Ly6G^–^, M-MDSCs). MDSCs have become the focus of intense study in cancer contexts, as they were originally described as a heterogeneous population with immunosuppressive functions in tumour-bearing hosts (Bronte 2009; Gabrilovich and Nagaraj 2009; Ostrand-Rosenberg and Sinha 2009; Guo et al. 2016; Groth et al. 2019). They promote the differentiation of Th17 cells and accelerate a variety of Th17 cell-mediated autoimmune diseases, such as systemic lupus erythematosus (SLE) (Wu H et al. 2016). Collagen-induced arthritis (CIA) is alleviated by MDSCs depletion, which also decreases Th17 cells numbers and serum IL-17A levels without influencing Treg cells (Zhang H, Wang, et al. 2015). In the murine model of multiple sclerosis (MS), known as experimental autoimmune encephalomyelitis (EAE), MDSCs proliferate and induce Th17 cells differentiation, thus playing an important role in this model. These findings indicate that MDSCs accumulation *in vivo* may in fact not exhibit suppressive activity and may even aggravate disease. The dysfunction of MDSCs promotes Th17 cells polarization and IL-17 production under the inflammatory microenvironment, substantially may be a factor driving autoimmune inflammatory pathology. Indeed, MDSCs do not alleviate inflammation but aggravate EAE and tissue damage by promoting Th17 cells development (Yi et al. 2012).

Th17 cells are a crucial subset of activated CD4^+^ T cells. Naïve CD4^+^ T cells can differentiate into pathogenic Th17 cells upon stimulation by cytokines such as IL-6 and IL-1β (Lee et al. 2020). Th17 cells produce IL-17A, which is involved in the pathogenesis of multiple inflammatory and autoimmune diseases such as MS, rheumatoid arthritis and colitis (Gao et al. 2010; Liu et al. 2011). Differentiation into M-MDSCs is promoted by IL-1β, and M-MDSCs promote IL-17 production by Th17 cells (Dumont et al. 2019). As MDSCs, especially M-MDSCs, are increased at the onset of EAE, reducing the accumulation of dysfunctional MDSCs at sites of inflammation may alleviate EAE. This may be related to Th17 cells inhibition and reduction of IL-17A levels (Yi et al. 2012; Zhang H, Huang, et al. 2015; Zhang H, Wang, et al. 2015; Ji et al. 2016; Wu H et al. 2016). The understanding of the relationship between MDSCs and Th17 cells has provided new perspectives on the treatment of EAE.

**Figure 1.**
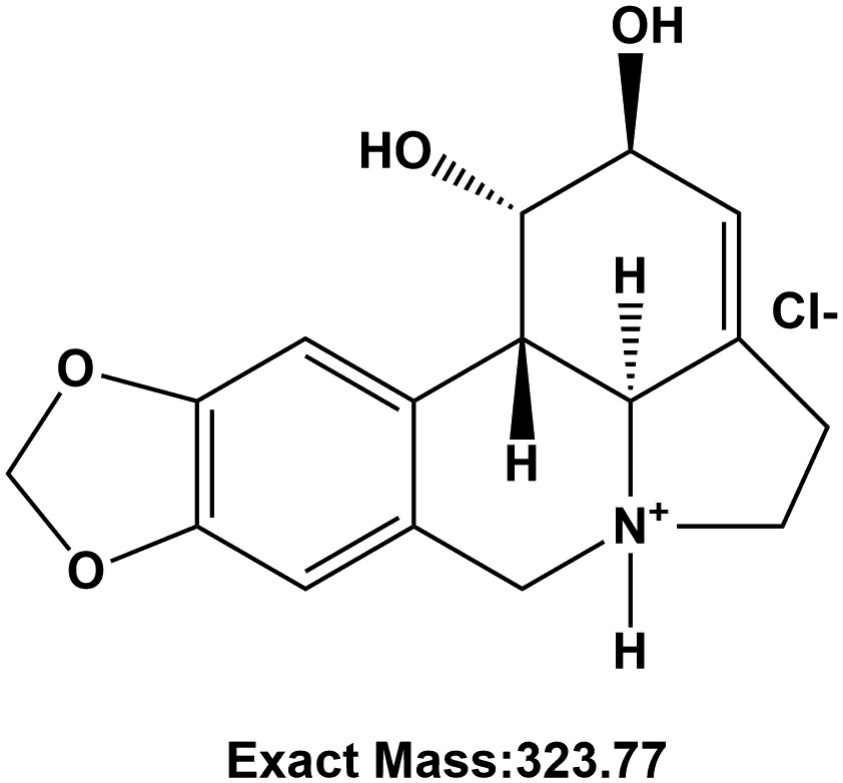
Chemical structure of pseudolycorine chloride.

Pseudolycorine chlorine (PLY) is an alkaloid isolated from *Narcissus tazetta* L. var. *Chinensis* Roem (Amaryllidaceae) (Figure 1). Recently, a structural analogue of pseudolycorine, lycorine, was shown to exhibit anti-tumour activity via the JAK/STAT pathway (Kang et al. 2012; Hu H et al. 2019), and anti-proliferative and pro-apoptotic activities in colorectal cancer (Wu S et al. 2018). STAT3 phosphorylation leads to the transcription of downstream genes that play critical roles in tumorigenesis and inflammation. The expression of myeloid-specific growth factors and inflammatory cytokines responsible for MDSCs differentiation depends on the STAT3 signalling pathway (Kang et al. 2012; Hu M et al. 2015; Ge et al. 2020). Therefore, MDSCs function and differentiation may be related to STAT3 signalling. However, the effect of PLY on EAE remains unclear.

Here, we performed *in vitro* and *in vivo* experiments to examine whether PLY has the potential to regulate MDSCs and, consequently, alleviate the progression of Th17 cell-mediated CNS autoimmunity.

## Materials and methods

### Mice

Female C57BL/6 (B6) mice were purchased from the Shanghai Model Organisms (Shanghai, China). All mice were fed normal food and water under specific pathogen-free (SPF) conditions in the Experimental Animal Center of Chengdu Medical College at consistent room temperatures (20–26 °C) and humidity (approximately 40–70%), with a 12 h light/dark cycle. All animal procedures were conducted in compliance with the protocol reviewed by the Animal Care and Use Committee of Chengdu Medical College (protocol number: CMC-IACUC-2021006).

### Reagents and antibodies

The following monoclonal antibodies (mAbs) against murine cell surface molecules were purchased from Biolegend (San Diego, CA): FITC-conjugated anti-CD11b (M1/70 clone), APC-conjugated anti-Ly6C (HK1.4 clone), PE-conjugated anti-Ly6G (1A8 clone), BV421-conjugated anti-Gr-1 (RB6-8C5 clone) and APC-conjugated anti-CD11b (M1/70 clone). The PE Annexin V Apoptosis Detection Kit with 7-AAD as well as the purified anti-CD4 (GK1.5 clone) and purified anti-Gr-1 (RB6-8C5 clone) antibodies were purchased from BD Pharmingen (San Diego, CA). Meanwhile, 5(6)-carboxyfluorescein diacetate succinimidyl ester (CFSE, 5 μM) and anti-IL-17 and anti-CD11b antibodies were purchased from Invitrogen (Carlsbad, CA) and Abcam (Waltham, MA), respectively. The following reagents were also purchased from Invitrogen (Carlsbad, CA): Alexa Fluor 594 goat anti-mouse IgG (H + L) and Alexa Fluor 488 goat anti-rabbit IgG (H + L). Myelin oligodendrocyte glycoprotein (MOG_35–55_) was purchased from Bankpeptide (Hefei, China). PLY was extracted from the bulbs of *N. tazetta* L. var. *Chinensis*, which were purchased in the spring of 2017 from Sanshenghuaxiang (Chengdu, China) and authenticated by one of the authors, Prof. Limei Li. The voucher specimen (no. LMNT1703) was in College of Pharmacy, Southwest Minzu University. The molecular weight and purity of the extracted PLY were 323.77 and 99%, respectively. PLY was dissolved in normal saline and filtered. Solcitinib and FK-506 (tacrolimus) were purchased from Selleck (Houston, TX) and CNSpharm (Houston, TX), respectively; both were dissolved in dimethyl sulphoxide.

### MDSCs differentiation

MDSCs from the bone marrow of 8–12-week-old C57BL/6 female mice, which were euthanized by cervical disconnection, and cultured in 24-well plates (1 × 10^6^ in 1 mL) containing RPMI 1640 (Gibco, Carlsbad, CA), supplemented with 10% FBS (Gibco, Carlsbad, CA), 1% penicillin and streptomycin (Hyclone, Logan, UT) and 20 ng/mL GM-CSF, 20 ng/mL IL-6 (PeproTech, Rocky Hill, NJ) for 48 h in the presence or absence of different concentrations of PLY or solcitinib (Selleck, Houston, TX). MDSCs were stained with FITC-anti-CD11b, BV421-anti-Gr-1, PE-anti-Ly6G and APC-anti-Ly6C and analysed using a Novocyte flow cytometer (ACEA Bioscience, Hangzhou, China). Cell culture was performed at 37 °C (5% CO_2_).

### MDSCs proliferation and survival

Isolated MDSCs were cultured in 96-well plates (2 × 10^5^ in 200 µL) containing RPMI 1640 (Gibco, Carlsbad, CA) supplemented with 10% FBS (Gibco, Carlsbad, CA), 1% penicillin and streptomycin (Hyclone, Logan, UT), 20 ng/mL GM-CSF, 20 ng/mL IL-6 (PeproTech, Rocky Hill, NJ) and 5 μM/L CFSE (Invitrogen, Carlsbad, CA) for 96 h in the presence or absence of different concentrations of PLY or solcitinib (Selleck, Houston, TX). After 48 h, MDSCs were stained with 7-AAD and PE-Annexin V and analysed with a Novocyte flow cytometer (ACEA Bioscience, Hangzhou, China). Cell culture was performed at 37 °C (5% CO_2_).

### Induction and assessment of EAE

MOG_35–55_ and complete Freund’s adjuvant (CFA, Chondrex, Woodinville, WA) were mixed at a ratio of 1:1 (v/v) to form a water-in-oil emulsion, and 200 μg/mouse was subcutaneously injected into 8–12-week-old C57BL/6 female mice. The mice were injected with pertussis toxin (PTX, 200 ng/mouse, Sigma-Aldrich, St. Louis, MO) at 0 and 48 h. From day 2, PBS, 40 mg/kg PLY, or 1 mg/kg FK-506 (tacrolimus, CNSpharm, Houston, TX) were intraperitoneally injected once a day for 21 d. Clinical scores were made according to the Benson score: 0, no clinical signs; 1, paralysed tail; 2, loss of coordinated movement, hind limb paresis; 3, both hind limbs paralysed; 4, forelimbs paralysed; and 5, moribund.

### Histopathology and immunofluorescence

Twenty-one days after administration (d23), the mice were anaesthetized (2% pentobarbital sodium, 50 mg/kg), perfused with saline and 4% (w/v) paraformaldehyde, embedded in paraffin, and dissected. Then, pathological sections of the spinal cords were stained with haematoxylin and eosin (H&E) or Luxol fast blue (LFB) (Beyotime, Shanghai, China). Images were captured using an OLYMPUS BX63 (Tokyo, Japan).

On the same day (d23), the dissected spinal cords were embedded in optimum cutting temperature (OCT) compound (SAKURA, Torrance, CA). After freezing, the spinal cords were stained with anti-mouse Ly-6G/Ly-6C (Gr-1) (1:100; BD Pharmingen, San Diego, CA, RB6-8C5 clone) and anti-rabbit CD11b (1:1000; Abcam, Waltham, MA), or with anti-rabbit IL17A (1:200; Abcam, Waltham, MA) and anti-mouse CD4 (1:100; BD Pharmingen, San Diego, CA, GK1.5 clone), followed by staining with Alexa Fluor 594 goat anti-mouse IgG (H + L) (1:500; Invitrogen, Carlsbad, CA) and Alexa Fluor 488 goat anti-rabbit IgG (H + L) (1:2000; Invitrogen, Carlsbad, CA). Red and green fluorescent images were captured using the OLYMPUS BX63 (Tokyo, Japan).

### Statistical analysis

Data are expressed as mean ± standard error of the means. Differences between groups were analysed using repeated analysis of variance (ANOVA) and *post hoc* Bonferroni’s correction or nonparametric Kruskal–Wallis tests (non-Gaussian distribution) using PRISM software (version 8.0; GraphPad Software, La Jolla, CA). Statistical significance was set at *p< 0.05 and **p< 0.01.

## Results

### PLY inhibits differentiation into M-MDSCs

**Figure 2.**
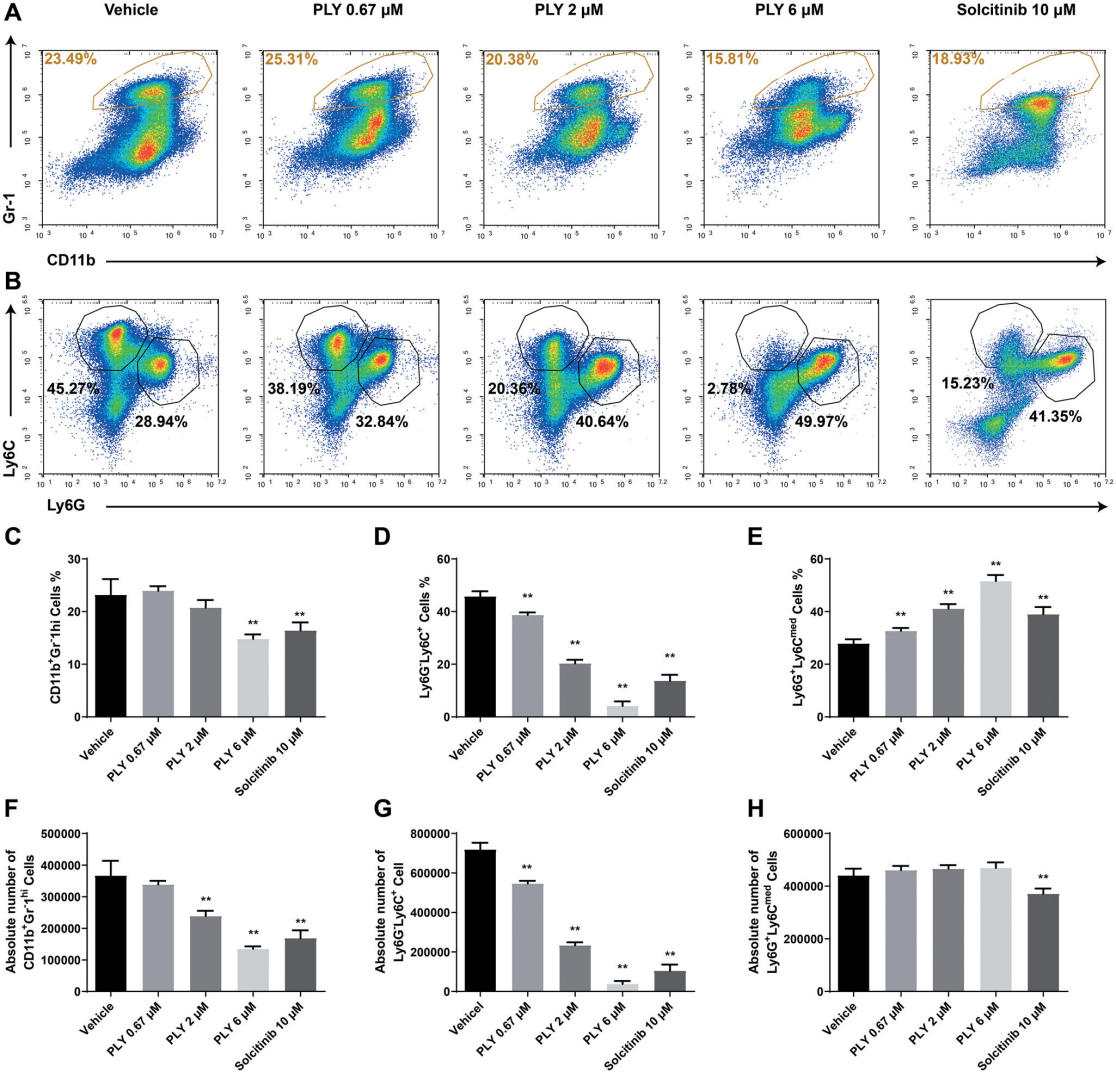
Effect of PLY treatment on GM-CSF and IL-6-derived MDSCs differentiation. The cells were stimulated with 20 ng/mL GM-CSF and 20 ng/mL IL-6, followed by treatment with PLY for 48 h at the indicated concentrations. Then, the cells were stained with anti-Gr-1, anti-CD11b, anti-Ly6C, anti-Ly6G antibodies and examined using flow cytometry. (A) The percentage of Gr-1^hi^CD11b^+^ MDSCs after treatment with 0.67, 2 or 6 μM PLY, PBS (vehicle), or solcitinib (positive control). (B) In the Gr-1^hi^CD11b^+^ MDSCs, two subsets were identified with anti-Ly6C and anti-Ly6G antibodies (Ly6G^−^Ly6C^+^ M-MDSCs; Ly6C^med^Ly6G^+^ G-MDSCs). The percentage of MDSCs (C), M-MDSCs (D) and G-MDSCs (E), and the absolute number of MDSCs (F), M-MDSCs (G) and G-MDSCs (H) are shown. One-way ANOVA was performed to determine statistical significance. The data are expressed as the mean ± standard error of the means of six independent experiments (**p< 0.01).

First, we investigated the effects of PLY, we treated MDSCs with 0.67, 2 and 6 μM PLY (Supplement figure 1), PBS (vehicle), and JAK-1 inhibitors (Gao et al. 2018; You et al. 2020) solcitinib (positive control) for 48 h. Specific M-MDSCs surface markers were examined using flow cytometry. We found that the number and percentage of Ly6G^−^Ly6C^+^ M-MDSCs after PLY treatment were significantly lower than those after vehicle treatment (Figure 2(A,D,G)). In contrast, the number of Ly6C^med^Ly6G^+^ G-MDSCs did not decrease after PLY treatment (Figure 2(A,H)), whereas the percentage of G-MDSCs increased (Figure 2(A,E)). Overall, the number and percentage of MDSCs were decreased after PLY treatment (Figure 2(A,C,F)).

### PLY has no effect on survival rate and apoptosis of MDSCs

**Figure 3.**
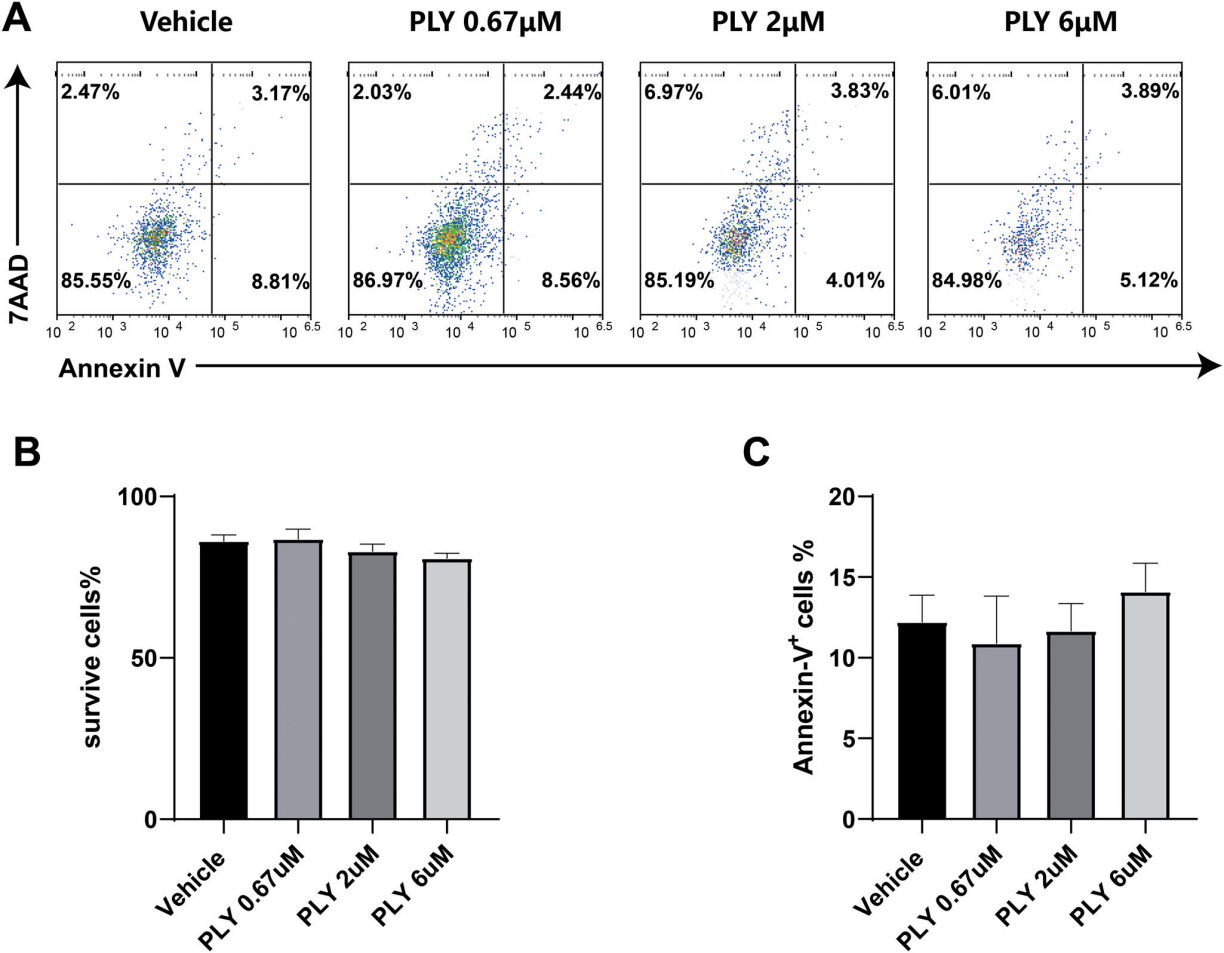
Effect of PLY treatment on the survival rate and apoptosis of GM-CSF and IL-6-stimulated MDSCs. The cells were stained with 7-AAD and annexin-V, and tested using flow cytometry. (A) After 48 h, MDSCs survival and apoptosis did not differ between the vehicle- and PLY-treated groups. The percentages of surviving MDSCs (B) and apoptotic (C) cells are shown. One-way ANOVA was performed to determine statistical significance. The data are expressed as the mean ± standard error of the means of six independent experiments (*p* > 0.05).

As the number of MDSCs decreased as the concentration of PLY increased, we examined whether PLY was cytotoxic. MDSCs were stimulated with 20 ng/mL GM-CSF and 20 ng/mL IL-6, followed by treatment with PLY for 48 h at the indicated concentrations. Then, we measured survival rate and apoptosis, and found no significant differences between the PLY- and vehicle-treated groups (Figure 3(A–C)). Thus, PLY did not affect the survival and apoptosis of MDSCs.

### PLY inhibits MDSCs proliferation *in vitro*

**Figure 4.**
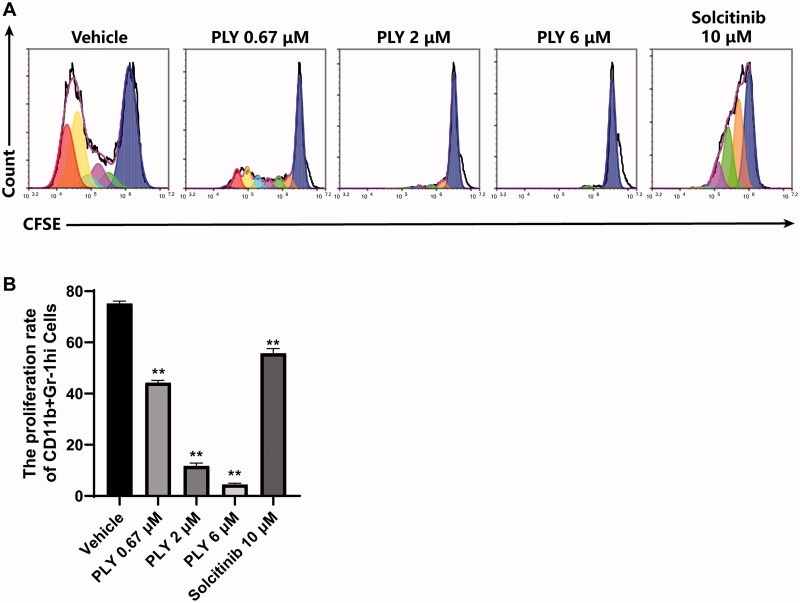
Effect of PLY treatment on the proliferation of GM-CSF and IL-6-stimulated MDSCs. GM-CSF and IL-6 were used to stimulate proliferation in the presence or absence of increasing concentrations of PLY. (A) After 96 h, 5(6)-carboxyfluorescein diacetate succinimidyl ester (CFSE)-stained cells were collected and examined using flow cytometry. (B) The proliferation rate of Gr-1^hi^CD11b^+^ MDSCs in the different groups. MDSCs proliferation was significantly inhibited by PLY and solcitinib. One-way ANOVA was performed to determine statistical significance. The data are expressed as the mean ± standard error of the means of six independent experiments (**p< 0.01).

The number of MDSCs plays an important role in the onset and development of inflammation. During inflammation, many pro-inflammatory and growth factors such as G-CSF, M-CSF, GM-CSF and IL-6 are produced in the microenvironment of the affected area. We found that PLY inhibited MDSCs expansion and differentiation into M-MDSCs (Figure 2) and that this effect was not due to cytotoxicity (Figure 3). Therefore, we investigated whether PLY could suppress MDSCs proliferation *in vitro*. Using CFSE, the proliferation of GM-CSF and IL-6-stimulated MDSCs was examined upon treatment with 0.67, 2 or 6 μM PLY; PBS (vehicle); or solcitinib (positive control). Compared with PBS, PLY inhibited MDSCs proliferation in three concentrations used (Figure 4(A)). Similarly, compared with solcitinib, medium- and high-dose PLY more effectively restrained MDSCs proliferation (Figure 4(A)). Thus, PLY significantly inhibited MDSCs proliferation.

### PLY reduces the number and percentage of M-MDSCs in EAE mice

**Figure 5.**
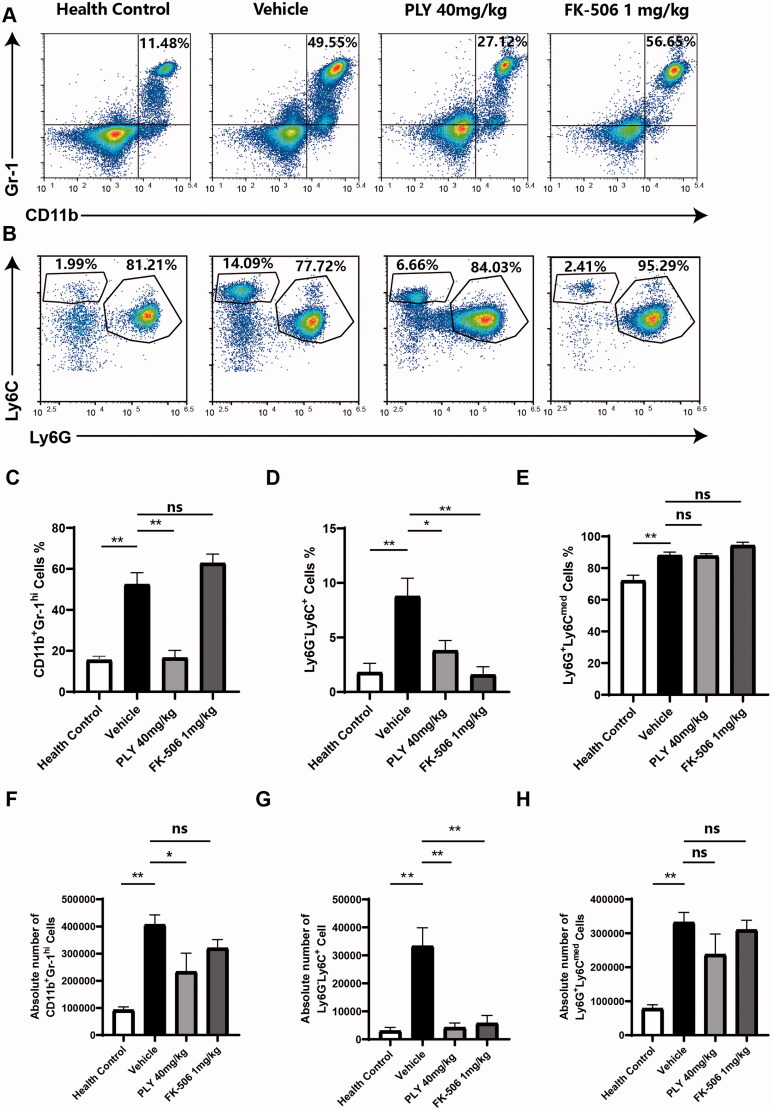
The number and percentage of MDSCs in EAE mice. After 21-day treatment with 40 mg/kg PLY, 1 mg/kg FK-506, or PBS, the peripheral blood of myelin oligodendrocyte glycoprotein (MOG_35–55_)-induced EAE mice was stained with anti-Gr-1, anti-CD11b, anti-Ly6C, anti-Ly6G antibodies, and examined using flow cytometry. The percentages of MDSCs (A) and G-MDSCs and M-MDSCs (B) in peripheral blood. Statistics showing the mean percentage of MDSCs (C), M-MDSCs (D), G-MDSCs (E) and the number of MDSCs (F), M-MDSCs (G) and G-MDSCs (H). One-way ANOVA was performed to determine statistical significance. The data are expressed as the mean ± standard error of the means (*n* ≥ 4 mice/group) (*p< 0.05, **p< 0.01).

MOG_35–55_-induced EAE is a good animal model for human MS. EAE mice were treated with 40 mg/kg PLY, PBS (vehicle) or 1 mg/kg FK-506 (tacrolimus, positive control), which inhibits the development of experimental allergic encephalomyelitis (Thomson et al. 1993; Gold et al. 2004), by intraperitoneal injection for 21 days. Next, we examined whether PLY affects M-MDSCs differentiation in EAE mice. All MOG_35–55_-induced EAE mice were sacrificed after treatment for 21 days and their peripheral blood was tested using flow cytometry. The number and percentage of MDSCs and M-MDSCs significantly decreased after treatment with PLY (Figure 5(A,C,D,F,G)). In addition, the percentage and number of Ly6G^+^Ly6C^med^ G-MDSCs were not significantly changed upon PLY treatment (Figure 5(E,H)). These results were similar to those obtained *in vitro* and demonstrated that PLY inhibits M-MDSCs differentiation in EAE mice.

### PLY treatment attenuates EAE severity

**Figure 6.**
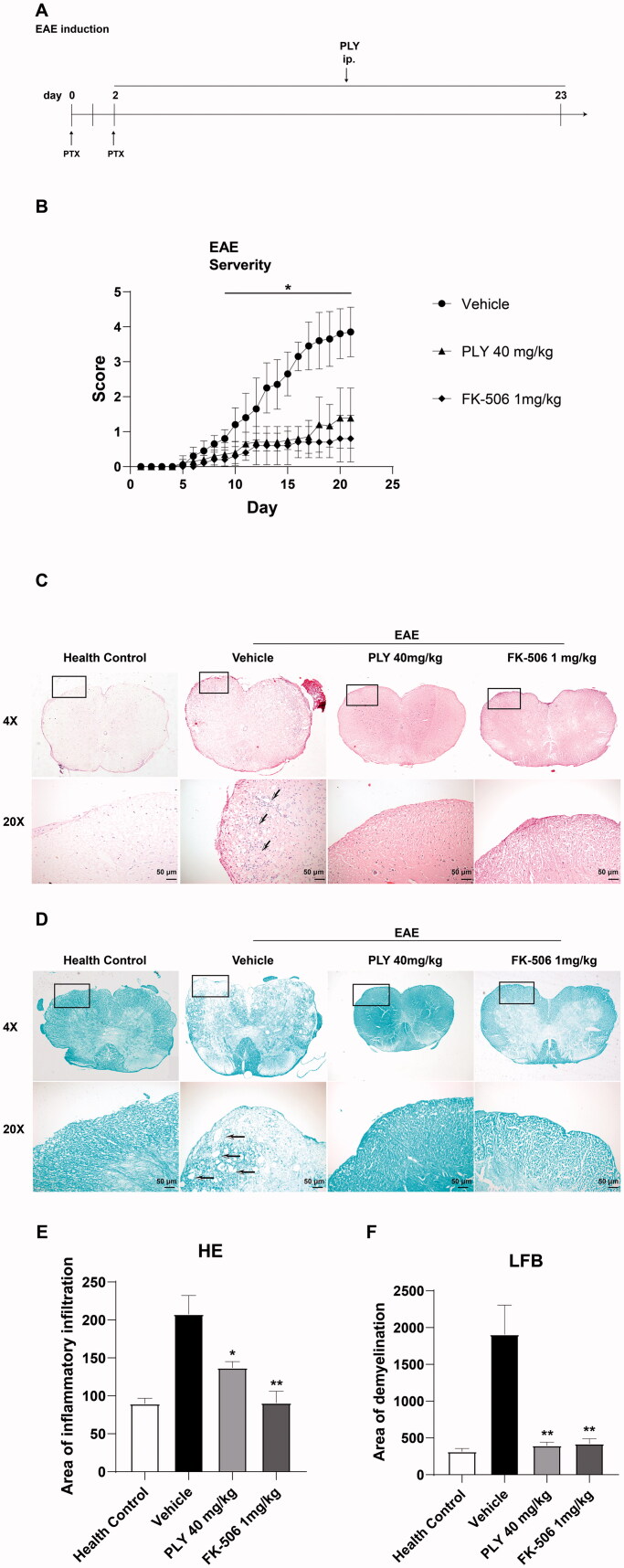
Pathology of EAE mice. EAE was induced by subcutaneous injection of 200 μg MOG_35–55_ emulsified in 50% complete Freud’s adjuvant (CFA). Individual mice were injected intraperitoneally with pertussis toxin (PTX) on day 0 and 2. PLY (40 mg/kg) and FK-506 (1 mg/kg) were intraperitoneally injected once a day from day 2 to 23. The severity of EAE was evaluated every day. (B) PLY and FK-506 significantly alleviated EAE. Kruskal–Wallis tests were performed to determine statistical significance (*n* = 5–10 mice/group) (*p< 0.05). (C) Haematoxylin and eosin (H&E) staining (×4 in the first row; ×20 in the second row) of the spinal cords from each group. Blue arrows represent inflammatory cells infiltration. (D) Luxol fast blue (LFB) staining (×4 in the first row; ×20 in the second row) of the spinal cords from each group. Bar graphs indicate the area of inflammatory infiltration (E) and demyelination (F). One-way ANOVA was performed to determine statistical significance. The data are expressed as the mean ± standard error of the means (*n* ≥ 3 independent experiments/group) (*p< 0.05, **p< 0.01).

We found that PLY and FK-506 alleviated EAE progression (Figure 6(A)). In addition, the severity of EAE in PLY-treated mice was similar to that in positive control mice. Meanwhile, H&E and LFB staining were performed to assess inflammation and demyelination, respectively. Compared with the vehicle, PLY and FK-506 reduced inflammatory infiltration and demyelination (Figure 6(B,C)). These results demonstrated that PLY alleviates the progression and severity of EAE and reduces inflammatory infiltration and demyelination.

### PLY inhibits MDSCs-dependent Th17 cells differentiation in the spinal cord of EAE mice

**Figure 7.**
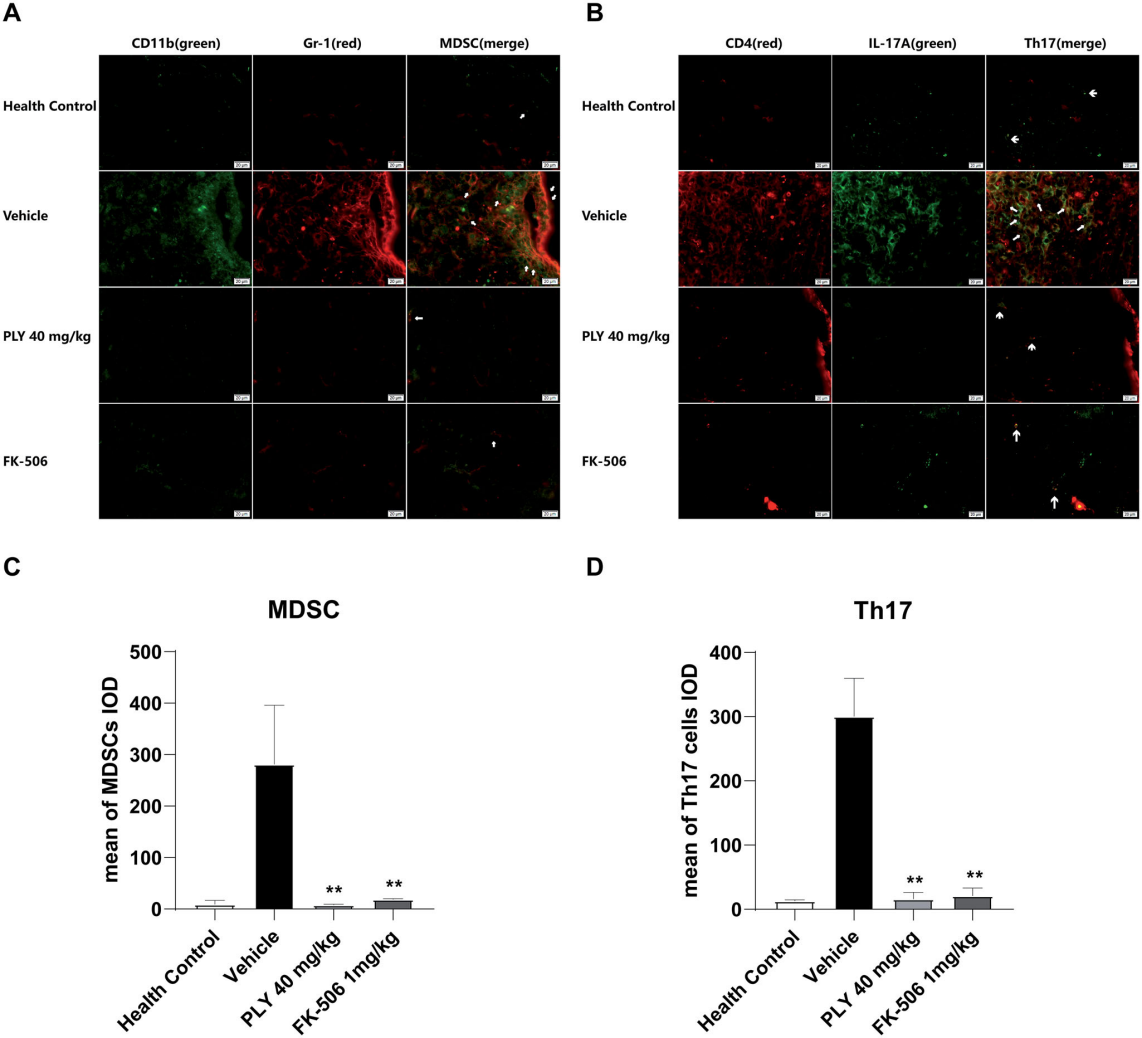
MDSCs and Th17 cells infiltration in the spinal cord of EAE mice. MDSCs were stained with anti-rabbit-CD11b and anti-mouse Ly-6G/Ly-6C (Gr-1); Th17 cells were stained with anti-rabbit-IL17A and anti-mouse CD4. Then, they were stained with Alexa Fluor 594 Goat anti-mouse IgG (H + L) and Alexa Fluor 488 Goat anti-rabbit IgG (H + L), respectively. (A) The left, middle and right panels represent CD11b (green), Gr-1 (red) and MDSCs (yellow), respectively, in the spinal cord. (B) The left, middle and right panels represent CD4 (red), IL-17A (green) and Th17 (yellow), respectively, in the spinal cord. The mean IOD (integral optical density) of MDSCs (C) and Th17 cells (D). MDSCs and Th17 cells infiltration into the spinal cord was significantly lower after PLY (40 mg/kg) or FK-506 (1 mg/kg) treatment than after vehicle treatment. One-way ANOVA was performed to determine statistical significance. The data are expressed as the mean ± standard error of the means (*n* ≥ 3 independent experiments/group) (**p< 0.01).

Th17 cells constitute the main pro-inflammatory T cell subtype in various autoimmune diseases. Previously, it was shown that MDSCs can inhibit Th17 cells differentiation and IL-17A secretion (Yi et al. 2012). Here, we found a positive correlation between MDSCs infiltration and IL-17A levels in the spinal cord of EAE mice (Figure 7). After treatment, the numbers of CD11b^+^Gr-1^hi^ MDSCs and Th17 cells in the spinal cords of each group were examined using immunofluorescence staining. The numbers of both cell types in the spinal cords of the PLY and FK-506 groups were lower than those in the spinal cords of the vehicle group (Figure 7). These data suggest that PLY can reduce the number of MDSCs, thereby inhibiting Th17 cells differentiation and IL-17A secretion.

## Discussion

PLY is a natural alkaloid extracted from *N. tazetta* L. var. *Chinensis*; its role is well established in cancer, but not in inflammatory diseases. In this study, we have shown that PLY inhibits MDSCs proliferation *in vitro* and ameliorates disease activity in EAE, a murine model of MS, through its effect on MDSCs. To our knowledge, this is the first report showing that PLY can alleviate EAE in mice and inhibit Th17 cells infiltration and differentiation by preventing MDSCs expansion and differentiation into M-MDSCs.

To understand the potential regulatory role of PLY on MDSCs, we used 0.67, 2 and 6 μM PLY to treat MDSCs and found that these concentrations can inhibit MDSCs differentiation *in vitro*. The number and percentage of MDSCs, especially M-MDSCs, significantly decreased after treatment, even though PLY was not cytotoxic at the concentrations used. Our data indicated that the percentage of surviving and apoptotic (Annexin-V^+^) cells did not significantly change after PLY treatment and demonstrated that PLY inhibits MDSCs proliferation.

MDSCs are a group of highly plastic immature immune cells that arise from common myeloid progenitors. MDSCs play an important role in the onset and progression of EAE. Here, we show that the number of MDSCs and Th17 cells in the spinal cord decreased with PLY treatment, which ameliorated inflammation and demyelination.

MDSCs function remains controversial. Under normal circumstances, immature myeloid cells differentiate into immune cells (e.g., dendritic cells, macrophages and mature granulocytes) (Groth et al. 2019); however, under abnormal conditions, such as autoimmune diseases, they can also differentiate into MDSCs (Condamine and Gabrilovich 2011). Thus, we consider that the numbers of MDSCs are largely increased under inflammatory conditions. Inflammation promotes the proliferation of various cells either indirectly or directly (Zhang S et al. 2018). Under continuous inflammatory conditions, early granulocyte or monocyte precursors differentiate into G-MDSCs and M-MDSCs, respectively. A large number of anti-inflammatory mediators are produced by MDSCs (Yang et al. 2008); however, MDSCs also participate in the inflammatory response by secreting pro-inflammatory factors (Chen et al. 2021). Similarly, chronic inflammatory disease leads to persistent pro-inflammatory signals, leading to inflammatory bone marrow microenvironments, myeloid cell expansion and MDSCs recruitment (Leimkuhler and Schneider 2019). Consistent with this, we also found a pathological increase in MDSCs under abnormal conditions.

Although the number of MDSCs increases in tumour and inflammatory microenvironments, MDSCs function may vary depending on the context. In the tumour microenvironment, the body is in a state of immunosuppression, and the function of immunocytes (e.g., T cells, B cells, dendritic cells and macrophages) is restrained. Some inflammatory factors, such as IL-6, GM-CSF and TNF-α, mainly promote tumour growth (Yang et al. 2008). Thus, the inhibitory effect of MDSCs may be dominant through their differentiation into tumour-associated macrophages, which can co-suppress NK, CD8^+^ and other immune cells (Zhang S et al. 2018). In contrast, MDSCs may play a pro-inflammatory role in a hyperactive inflammatory environment. Researchers generally believe that, compared to G-MDSCs, M-MDSCs are pro-inflammatory (Zhang H, Wang, et al. 2015; Chen et al. 2021). Some studies have found that the inhibitory function of M-MDSCs is damaged during the development of SLE in MRL/LPR mice and that the cells play a pro-inflammatory role in that context. By decreasing the levels of M-MDSCs-derived IL-1β, Th17 cells differentiation decreased, thereby alleviating the disease (Ji et al. 2016). In addition, MDSCs and the Arg-1 from M-MDSCs have been found to increase in primary membranous nephropathy, and MDSCs were shown to promote Th17 cells differentiation in an Arg-1-dependent manner (Li et al. 2020). These reciprocal and distinctive actions of MDSCs and Th17 cells collaboratively perpetuate multiple pathogenic processes, including aggravated inflammation. In a study by Guo et al. (2016), MDSCs decreased T cells inhibitory activity during the progression of arthritis. This may be determined by different inflammatory signals present in the inflamed sites, which are part of the functional plasticity of MDSCs.

Thus, we speculated that MDSCs inhibition could alleviate the inflammatory response in EAE through the inhibition of MDSCs-mediated Th17 cells differentiation; this would decrease the production of inflammatory cytokines such as IL-17A, which indirectly and directly damage myelin sheaths in the CNS (Elliott et al. 2018). Consistent with this, we observed an increase in the number of MDSCs as EAE progressed, whereas PLY treatment decreased the proportion and number of MDSCs and M-MDSCs, reduced MDSCs and Th17 cells infiltration into the CNS, and alleviated the disease.

Myelin-specific Th17 cells are responsible for the pathogenesis of EAE and MS. The number of MDSCs and Th17 cells decreased after PLY treatment in the spinal cord; however, naïve CD4^+^ T cells differentiate into Th17 cells in multiple ways. For example, naïve CD4^+^ T cells differentiation into non-pathogenic Th17 cells is stimulated by IL-6 and TGFβ, while its differentiation into pathogenic Th17 cells is stimulated by IL-16 and IL-1β (Chung et al. 2009; Das et al. 2009). IL-1β promotes the expansion of MDSCs and is associated with Th17 cells. However, we did not use IL-1β in our study because Th17 cells differentiation can also be regulated by STAT1/3/5 (Damasceno et al. 2020). IL-1β accelerates production of MDSCs mainly via the activation of NF-κB or AP-1 (Wang et al. 2020).

However, IL-6 and GM-CSF mainly stimulate the JAK-STAT pathway. In addition, the inhibition ability of M-MDSCs was improved by increasing the levels of reactive oxygen species through STAT3 activation (Pan et al. 2016). The proliferation of neutrophil T cells is restrained by the action of GM-CSF on M-MDSCs via the JAK-STAT pathway. JAK-2/STAT-3 inhibition was shown to reduce the number of MDSCs, and these pathways are critical for MDSCs proliferation in cancer (Bayne et al. 2012; Nishimura et al. 2015; Sendo et al. 2019). Thus, we used IL-6 and GM-CSF to co-stimulate MDSCs and speculate that IL-6 and GM-CSF play important roles in MDSCs proliferation and differentiation via JAK-STAT signalling; moreover, these pathways may be associated with targets of PLY.

## Conclusions

Our data indicated that PLY alleviates EAE through its ability to target a range of anti-inflammatory pathways, including (a) inhibition of MDSCs proliferation and differentiation into M-MDSCs, (b) inhibition of inflammatory infiltration and demyelination in the spinal cord and (c) prevention of MDSCs and Th17 cells migration into the CNS. Future studies are needed to clarify the mechanism whereby PLY alleviates EAE. Collectively, our findings provide new insights into the importance of MDSCs in autoimmune diseases and may help to design new therapies for MS, as they suggest that PLY may be an excellent candidate drug for MS treatment.

## Supplementary Material

Supplemental Material
