# Supplementary figures and images for: Pseudolycorine chloride ameliorates Th17 cell-mediated central nervous system autoimmunity by restraining myeloid-derived suppressor cell expansion
Source: Pharm Biol. 2022 Sep 9;60(1):899–908. doi: 10.1080/13880209.2022.2063344 (PMC9467541; doi:10.1080/13880209.2022.2063344)

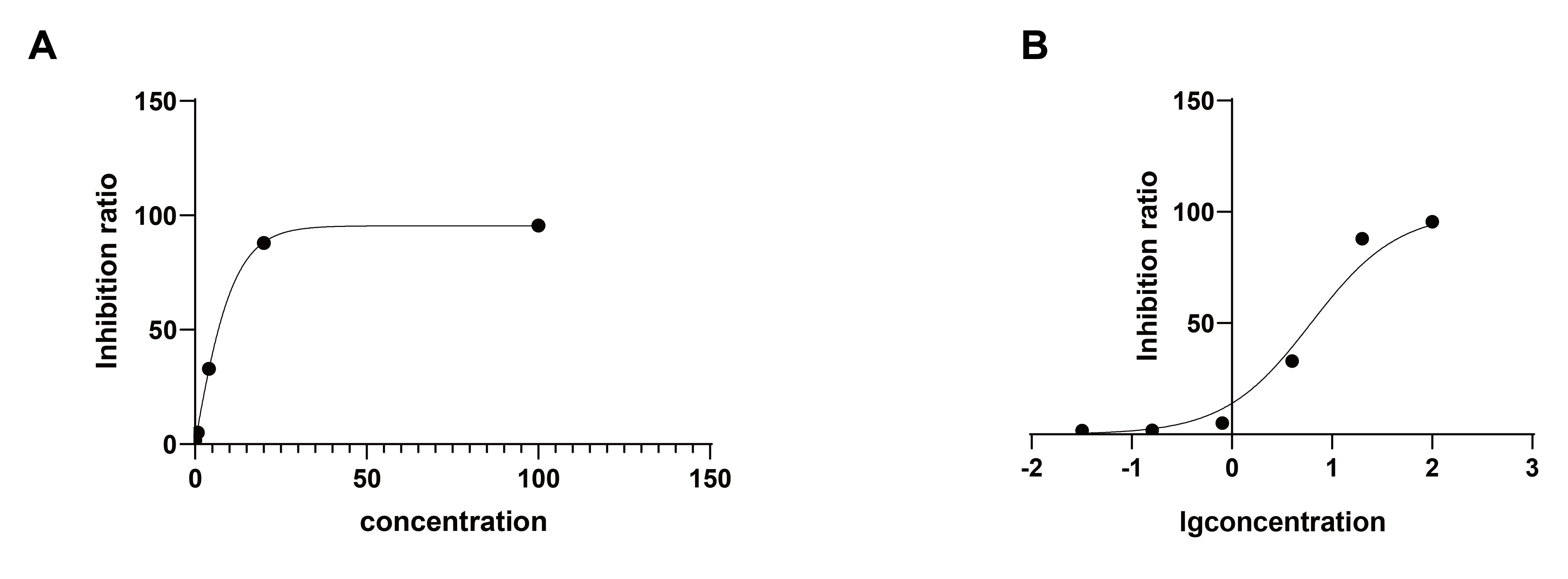

Supplement: Supplemental Material [file IPHB_A_2063344_SM5000.tif]
